# Molecular Dynamics Simulations of Claudin-10a and -10b Ion Channels: With Similar Architecture, Different Pore Linings Determine the Opposite Charge Selectivity

**DOI:** 10.3390/ijms25063161

**Published:** 2024-03-09

**Authors:** Santhosh Kumar Nagarajan, Jörg Piontek

**Affiliations:** Clinical Physiology/Nutritional Medicine, Department of Gastroenterology, Rheumatology and Infectious Diseases, Charité—Universitätsmedizin Berlin, Corporate Member of Freie Universität Berlin and Humboldt-Universität zu Berlin, 12203 Berlin, Germany; santhosh-kumar.nagarajan@charite.de

**Keywords:** claudin, ion channel, tight junction, molecular dynamics simulation, assembly, paracellular permeability, charge selectivity

## Abstract

Claudin polymers constitute the tight junction (TJ) backbone that forms paracellular barriers, at least for bigger solutes. While some claudins also seal the barrier for small electrolytes, others form ion channels. For cation-selective claudin-15 and claudin-10b, structural models of channels embedded in homo-polymeric strands have been suggested. Here, we generated a model for the prototypic anion-selective claudin-10a channel. Based on previously established claudin-10b models, dodecamer homology models of claudin-10a embedded in two membranes were analyzed by molecular dynamics simulations. The results indicate that both claudin-10 isoforms share the same strand and channel architecture: Sidewise unsealed tetrameric pore scaffolds are interlocked with adjacent pores via the β1β2 loop of extracellular segment 1. This leads to TJ-like strands with claudin subunits arranged in four joined rows in two opposing membranes. Several but not all *cis*- and *trans*-interaction modes are indicated to be conserved among claudin-10a, -10b, and -15. However, pore-lining residues that differ between claudin-10a and -10b (i.e., R33/I35, A34/D36, K69/A71, N54/D56, H60/N62, R62/K64) result in opposite charge selectivity of channels. This was supported by electric field simulations for both claudins and is consistent with previous electrophysiological studies. In summary, for the first time, a structural and mechanistic model of complete and prototypic paracellular anion channels is provided. This improves understanding of epithelial paracellular transport.

## 1. Introduction

Paracellular permeability in epithelia and endothelia is regulated by tight junctions (TJs) in a size- and charge-selective manner. TJs are protein complexes that contain a multitude of membrane-associated scaffolding (e.g., ZO-1), signaling, and transmembrane proteins (mainly claudins, TAMPs, and JAMs) [1,2]. The members of the claudin family of tetraspan membrane proteins (Figure S1) form the backbone of TJ strands. They constitute both barriers as well as channels that directly regulate paracellular permeability [3,4,5,6]. Functionally, claudins can be roughly grouped into (i) barrier-forming claudins (such as CLDN1, -3, -5, and -11) that almost completely block solute permeation, (ii) channel-forming claudins (such as CLDN2, -10a, -10b, -15, and -17) that form size- and charge-selective channels, and (iii) context-dependent claudins (such as CLDN4, -7, -8, and -16), whose permeability properties strongly depend on additional TJ components and conditions [1]. In addition, claudins can be grouped based on sequence similarity into classic (typical) and non-classic (atypical) claudins [7]. Classic claudins contain common sequence motifs that were shown to be critical for claudin self-assembly into polymeric TJ-like strands [4,8,9,10,11,12]. Non-classic claudins largely lack these motifs, and for most of them, strand incorporation depends on co-oligomerization with classic claudins [4,13,14]. Due to the conserved motifs, classic claudins were proposed to share a similar polymerization mechanism and, thus, a similar overall strand architecture [4,12,15]. However, this concept needs further validation. Claudins polymerize via *cis*-interactions between polypeptide chains (subunits) located within one membrane and *trans*-interactions between subunits in two opposing membranes at cell–cell contacts (Figure S2) [7]. Hetero-oligomerization between different claudin subtypes is possible for compatible claudin subunits only [4,13,14,16,17]. 

Different architectural models have been suggested for CLDN15 and other classic claudins [4,10,12,18,19,20,21,22,23]. However, the joined double rows (*JDR*) model, first introduced by Suzuki et al. [10] and later refined and expanded by others, is supported best by experimental and modeling studies [4,12,15,24,25,26,27]. According to this model, claudin subunits interact via (i) a face-to-face *cis*-interface and (ii) a linear *cis*-interface, resulting in an antiparallel double-row of subunits within one membrane. *Trans*-interactions between subunits join the two double rows of opposing cell membranes into functional strands (Figures S2). During assembly, claudins were proposed to form small *cis*-oligomers before *trans*-interaction at cell–cell contacts, which triggers *cis*-/*trans*-polymerization [12].

Recently, we compared two variants of Suzuki’s *JDR* architectural model for the two related cation channel-forming CLDN10b and -15: a *tetrameric-locked pore barrel* vs. *octameric-interlocked pore barrels* model [15]. Molecular dynamics simulations of membrane-embedded dodecamers indicated that CLDN10b and CLDN15 share the same architecture of TJ-strands: Octameric *Interlocked pore Barrels* (*IB*) in which sidewise-unsealed tetrameric pore scaffolds are interlocked with adjacent pores by the β1β2 loop of the extracellular segment (ECS) 1 (Figure S2B,C). On the one hand, this loop mediates hydrophobic clustering and, together with ECS2, *cis*- and *trans*-interaction between subunits of adjacent tetrameric pore scaffolds, leading to the formation of polymeric strands. On the other hand, the β1β2 loop contributes to the lining of the pathway for ion conduction and, thus, to selectivity properties [15]. 

For paracellular anion channels, no similar oligomer modeling and MD simulations have been performed so far. Simulations and free energy calculations have been performed for CLDN4 [21,28,29]. However, they were restricted to tetramers and did not consider embedment of channels into strands, and the contribution of CLDN4 to anion channels is debatable [2,30,31]. In addition, a pathogenic CLDN5 mutant was reported to form an anion channel [32], and corresponding elegant MD simulations and free energy calculations supported this conclusion [33]. However, the physiological function of CLDN5 is to form a barrier, in particular at the blood–brain barrier [1,7,32,34]. Thus, we aimed here to analyze the prototypic and functionally well-characterized anion channel formed by CLDN10a [35,36,37]. For this purpose, we took advantage of the high sequence similarity between CLDN10a and CLDN10b—analyzed previously. The two isoforms differ only in transmembrane helix 1 (TM1) and ECS1, and the differing charge selectivity is mediated by ECS1 [38], which still shows ~57% sequence similarity between both isoforms.

Here, we generated and tested a CLDN10a dodecamer (three-pore) model based on the previously generated CLDN10b model and the previously established workflow, including homology modeling, membrane embedment, distance constraints, and Desmond MD simulations. The results indicate that CLDN10a and -10b channels share a common interlocked pore barrels *JDR* architecture. Nevertheless, next to conserved interaction motifs, some inter-subunit interfaces differ partly. In addition, pore-lining residues differ between CLDN10a and -10b channels and result in opposite charge selectivity.

## 2. Results

### 2.1. Generation of Dodecamer Model for CLDN10a 

Previously, we generated the *Iinterlocked pore Barrels* (*IB*) model of CLDN10b channels based on experimental support [12,39], the CLDN15 *JDR* strand model [10], homology modeling, and MD simulations [15]. This dodecamer model consists of three adjacent interlocked pore tetramers with the middle pore stabilized and completely closed sidewise by residues of eight subunits (chains) (Figure S2B,C). Here, we generated a CLDN10a homology model using the CLDN10b *IB* model as a template. The rationale was the assumption that CLDN10a and -10b share a similar overall architecture due to the high sequence similarity (80% in the template region, Figure S1A), a similar fold prediction (Figure S1B), and similar function (paracellular ion channel). 

The CLDN10a protein dodecamer was embedded in two POPC lipid bilayers, and NaCl and water were then added. The system was relaxed and equilibrated with gradually releasing constraints similar to those established previously [15] and described in the Methods and Figure S3. Five variant MD simulation production runs (each for 100 ns) of the interlocked pore barrels (IB) model were performed and compared: 

*IB-1* (*Interlocked pore Barrels* model 1): The assumed face-to-face interface was slightly supported by a force constant of 1 kcal mol^−1^ Å^−2^ on the backbone of cysteine 61 (C61) in β4-strand. All other atoms in the system could move freely. In addition, the initial *IB-1* model was refined further to increase the interface-wise symmetry of the model. Different constraints during the production runs led to four variants of the refined model: *IB-2*—this model has the same C61 backbone constraints as *IB-1*; *IB-2+lic*—this model has additional constraints on the linear-*cis* interface (backbone and β-carbon of I67 and F68, 1 kcal mol^−1^ Å^−2^); *IB-2+hc*—this model has additional constraints on the backbone of helices (1 kcal mol^−1^ Å^−2^); and *IB-2+ohc*—this model has additional constraints on the backbone of helices of the four outer subunits, i.e., subunits that do not line the middle pore (1 kcal mol^−1^ Å^−2^).

**Figure 1:**
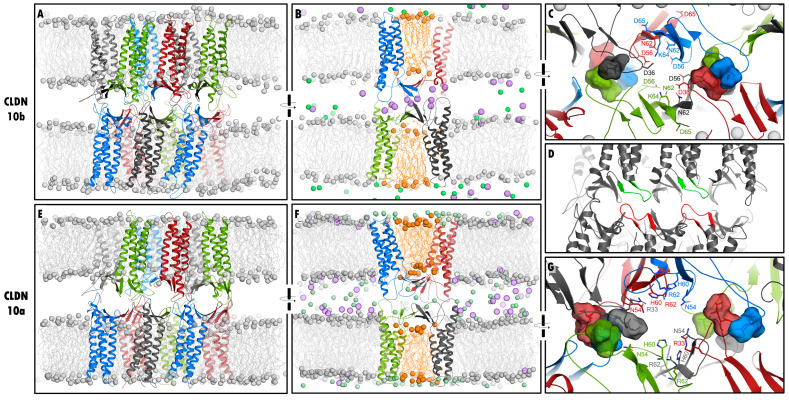
Comparison of CLDN10b and CLDN10a dodecamer (three pore) models. (**A**–**C**) Snapshots of the production run at 100 ns for the CLDN10b model (CLDN10b_8IBno, taken and modified from [15]). (**A**) Front view; (**B**) turned view on the middle pore, claudin subunits (chains) shown as a colored cartoon, POPC lipids as lines, and phosphate headgroups as spheres. (**E**–**G**) Snapshot of the production run at 100 ns for CLDN10a model (IB-2): (**E**) front view; (**F**) turned view on the middle pore. Protein chains were embedded well in membranes, and the overall arrangement, including trapped lipids (orange) between the two claudin rows, was similar to that of CLDN10b. In the CLDN10a pore, mainly Cl^−^ ions (green spheres) were present, whereas in that of CLDN10b, mainly Na^+^ ions (magenta spheres) were present, fitting to opposite charge selectivity of the two different channels. (**D**) Schema, showing the contact of β1β2 loops (red and green) between *trans*-interacting subunits (other claudin parts gray), highlighting the kinked/flat orientation of the loops towards subunits in the same membrane. This results in interlocked loops of neighboring pores (interlocked barrels (IB) arrangement). CDLN10a and -10b models showed similar IB arrangements. (**C**,**G**) Close-up of dodecamer centers. On both sides of the middle pore, hydrophobic clusters were formed similarly for CLDN10a and -10b by V37/I38 (CLDN10a) and V39/I40 (CLDN10b) residues (shown as surfaces) of *trans*- and *cis*-interacting β1β2 loop tips from four subunits (blue, green, red, and black). In contrast, hydrophilic pore-lining residues (sticks) differed strongly between CLDN10a and -10b. See also Video S1.

The *IB-2* model variant was selected as the reference model, and, as an example, the 100 ns snapshot of the corresponding MD simulation is shown in Figure 1E–G. For all simulated model variants (*IB-1*, *IB-2*, *IB-2+lic*, *IB-2+hc*, and *IB-2+ohc*), all twelve CLDN10a subunits were embedded well in the two opposing membranes (hydrophobic transmembrane helices were covered with acyl chains of intact (water-free) lipid bilayer throughout the simulations). The overall arrangement of the CLDN10a dodecamer was similar to the *JDR*-like arrangement of CLDN10b, including interlocked barrel-like pores and lipids trapped in the central region between the two claudins rows of one membrane (Figure 1A–G). Compared to the pre-equilibrated starting structures and to the CLDN10b model, all interface types (including face-to-face-cis, linear-cis, and ECS2-ECS2-trans interfaces) were largely maintained. Also similar to CLDN10b, in CLDN10a dodecamers, stable clusters of the hydrophobic tips of β1β2 loops from four subunits were formed between neighboring pore centers. The latter were lined by hydrophilic residues (Figure 1C,G). However, the pore-lining residues differed strongly between the two claudin-10 isoforms. Of note, individual interfaces varied in detail over time between the individual subunits and between the five simulation lines (*IB-1*, *IB-2*, *IB-2+lic*, *IB-2+hc*, and *IB-2+ohc*). Thus, we evaluated and compared the MD simulations of the CLDN10a model variants in detail. 

### 2.2. Evaluation of the CLDN10a Dodecamer Models

#### 2.2.1. RMSD Indicates Overall Stability of CLDN10a Dodecamer Models 

The stability of the individual subunits and of the oligomer during the production run was evaluated by calculating the root-mean-square-deviation (RMSD) of the protein backbone. The RMSD for the entire dodecamer with respect to its initial structure increased steadily for IB-1, whereas it reached a plateau for all IB-2 model runs after ~90 ns (Figure S4A). It was lowest (~1.0 Å) for the strongest constrained one (IB-2+hc) and highest (~2.2 Å) for the run with constraints on the linear-*cis* interface (IB-2+lic). For IB-2 and IB-2+ohc, it showed an intermediate RMSD of ~1.7 Å. These relative constant values were in the range of CLDN10b dodecamer models (1.5–2.2 Å, Figure S4A) and slightly lower than the RMSD for CLDN15 dodecamer simulations (<3.5 Å, [26]). This indicated the overall stability of the membrane-embedded CLDN10a oligomers, which was most relevant for the weakly constrained IB-2 model variant.

In addition, RMSD was calculated with respect to the initial structure of each dodecamer subunit (Figure S4B). The RMSD and, thus, structural change over time differed between individual subunits, reflecting a certain heterogeneity within the dodecamers. For each model variant, the mean RMSD of all subunits reached a plateau after ~70 ns with values (≤2 Å) in a range similar to that obtained in CLDN15 polymer simulations [27]. The RMSD values of the transmembrane helices backbone with respect to the initial structure were slightly below the values of the whole protein backbone for each model variant, with mean RMSD values of ~ 1.5 Å throughout the last 30 ns for IB-1, IB-2, IB-2+lic, and IB-2+ohc and ~ 1.2 Å for IB-2+hc (Figure S4C). 

#### 2.2.2. Definition and Comparison of Interfaces within Dodecamer by Residue Contact Maps: *cis*- and ECS2-*trans*- Interfaces

To define the interfaces between the subunits in the dodecamer, residue contact maps were generated for the regions of all subunits that are relevant to the *JDR* architecture model [10,15]. 

**Figure 2:**
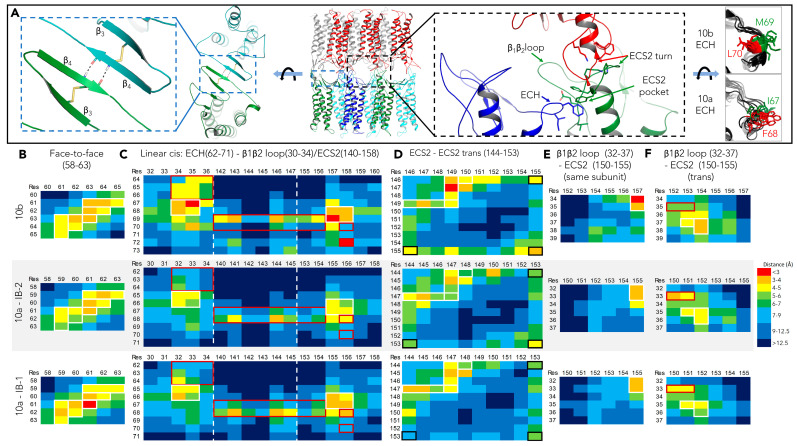
Inter-subunit residue contact maps for CLDN10b and CLDN10a models. (**A**) Overview of different inter-subunit interfaces. Middle: Front view on CLDN10a dodecamer with subunits as colored cartoons. Left: Turned view on ECS1+2 of two bottom subunits (dashed blue box in the middle) highlighting face-to-face cis interface with H-bonds between β4 strands. Right: Close-up of the dashed black box in the middle highlighting the linear-cis interface between the ECH region (blue) and ECS2 pocket (green) and *trans*-interface between two ECS2 turn regions (green, red). Relevant residues are shown as sticks. Far right: Comparison of CLDN10a and CLDN10b ECH regions. Overlay of several subunits showing different orientations of F68/I67 in CLDN10a and of corresponding L70/M69 in CLDN10b model. (**B**–**F**) Mean distances (closest atoms) between the numbered residues of protein region pairs reflecting the different interface types. For each interface type, the multiple individual interfaces in the dodecamer were averaged. Shown are interaction-relevant parts of contact maps categorized according to interface types: (**B**) face-to-face-cis, (**C**) linear-cis, (**D**) ECS2-ECS2-trans, and (**E**,**F**) β1β2 loop-ECS2. In headlines, corresponding residue numbers of CLDN10a are given in brackets. Residue contact pairs are boxed in white if similar, in red if shifted, and in black when different between CLDN10a and -10b. The contact maps provide interface fingerprints for comparison of the different claudin models. CLDN10b data taken from previous simulations [15].

(i) First, the face-to-face-*cis* interface was analyzed. Along the pore pathway, the bottom and top of the pore are each formed by two claudin subunits connected by this antiparallel association of ECS1 β4-strands (Figure 2A and Figure S2A). At this interface, for CLDN10b, close distances (<5 Å) were found between the β4-strand residues S61 to D65 with the pair C63–C63 in the center (data taken from previous simulations [15], Figure 2B). Similarly, for CLDN10a, the corresponding residues F59 to P63 were close to each other, with the C61–C61 pair in the center. The mean distance for CLDN10a-*IB-1* was slightly lower than for -*IB-2* (Figure 2B). In sum, face-to-face *cis*-interfaces were similarly formed for the CLDN10a and -10b dodecamers.

(ii) Within one claudin row (of *JDR*-like polymers), neighboring subunits are connected via the ECH region of ECS1 and ECS2 (linear-*cis* interface, Figure 2A and Figure S2 [10,15]). For this interface, in CLDN10a models (*IB-1*/-*IB-2*), close proximity of ECH region with ECS2 pocket and base of β1β2-loop was found, similar to CLDN10b: T66/S68 (corresponding 10a/10b residues) was close to E155/E157 and F65/P67, T66/S68 close to T32/T34, R33/I35 (Figure 2C). Also, similar to CLDN10b, a residue of the hydrophobic ECH region was close to the hydrophobic ECS2 pocket formed by T141/T143, F144/F146, F145/F147, L156/L158. However, for CLDN10a, instead of I67 corresponding to M69 of CLDN10b, F68 corresponding to L70 of 10b was predominantly sticking to the pocket (close distances, Figure 2C). This difference between CLDN10a and -10b was at least partly due to the non-α-helical conformation of the region T66-A71 corresponding to the α-helical ECH region S68-D73 of CLDN10b (Figure 2A right). 

Also, V70 of CLDN10a was further away from the pocket (L156/L158) than the corresponding L72 of CLDN10b. Furthermore, in CLDN10a, R62, P63, H64 were more distant from T32, R33, A34 than the corresponding K64, D65, F66 from T34, I35, D36 of 10b (Figure 2C), likely due to charge repulsion (R62-R33 in CLDN10a) instead of attraction (K64-D36 in CLDN10b). In sum, for both claudins, the linear-*cis* interface contained conserved hydrophobic and electrostatic components. However, due to the non-α-helical conformation of the ECH region, a variant hydrophobic fit was achieved for CLDN10a. 

(iii) Close to the pore entrance, each pore side is bounded by ECS2-ECS2 *trans*-interaction (ECS2-ECS2 *trans*-interface, Figure 2D, [10,15]). Here, proximities were also mainly similar for CLDN10a and -10b. Closest distances were found for (10a/10b residues) P147/P149 and F144/F146 or F145/F147, as well as F145/F147 and L148/L150. However, the pairwise distances, including those of K153/K155 with K153/K155 or F144/F146, were slightly larger for CLDN10a (Figure 2D)). 

(iv) Regarding β1β2 loop-ECS2 contacts, within one CLDN10a/-10b subunit, T32/T34, R33/I35 of β1β2 loop were close to E155/E157 in ECS2 for CLDN10a as well as for CLDN10b, indicating similar positioning of this β1β2 loop region relative to E155/E157 (Figure 2E). In *trans*, S35/G37, S36/T38 of β1β2 loop were close to E151/E153, Q152/Q154 in ECS2, similar to both CLDN10a and-10b. However, partly due to electrostatic attraction, R33, V150, and E151 were closer to CLDN10a than the corresponding I35, V152, and E153 in CLDN10b (Figure 2F).

The contact maps show a similar overall pattern for CLDN10b and -10a, reflecting a similar overall architecture of both channels. However, the pair distances are, on average, slightly higher for the CLDN10a dodecamer simulations *IB-1* and *IB-2*. This and differences in contact details are evaluated below. 

#### 2.2.3. Further Analysis of *cis*- and ECS2-*trans*- Interfaces in CLDN10a Dodecamers

The CLDN10a model variants (*IB-1*, *IB-2*, *IB-2+lic*, *IB-2+hc*, *IB-2+ohc*) were further analyzed with respect to their different interface types. 

**Figure 3:**
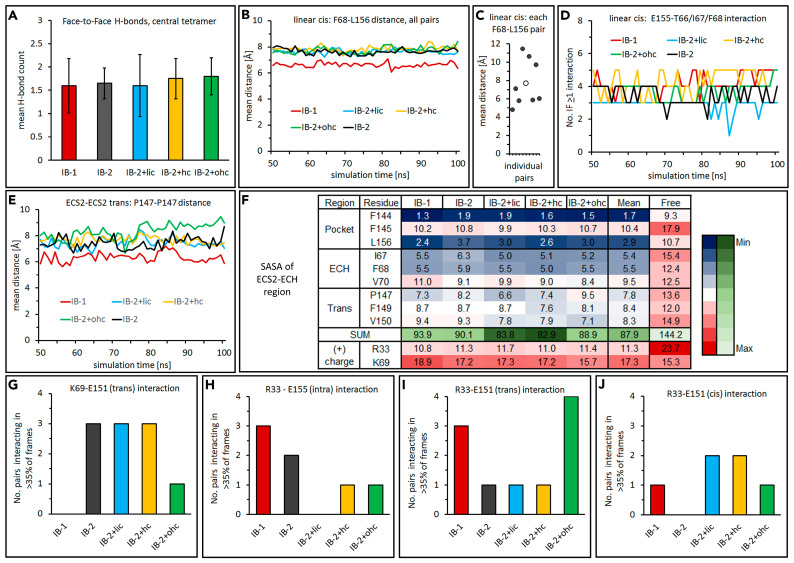
Detailed interface analysis for CLDN10a dodecamer model variants IB-1, IB-2, IB-2+lic, IB-2+hc and IB-2+ohc. The last 50 ns of production runs were analyzed. (**A**) Face-to-face-cis interface: F59 to P63 backbone H-bond counts per β4-β4-strand interface for the two interfaces of middle pore in dodecamer. Mean ± SD. (**B**) Linear-cis interface: F68(Cγ)-L156(Cγ) distances over time. Mean of eight pairs (interfaces) in dodecamer. (**C**) Linear-cis interface: Mean F68(Cγ)-L156(Cγ) distances in 50 ns for each of the F68-L156 pairs in dodecamer (black dots) and mean of the eight pairs (white dot). (**D**) Linear-cis interface: Number of interfaces (IF, out of eight) with ≥one E155-T66/I67/F68 (side and main chain) electrostatic interaction count over time. (**E**) ECS2-ECS2 *trans*-interface: P147(Cα)-P147(Cα) distances over time. Mean of four interfaces in dodecamer. (**F**) Solvent accessible surface area (SASA) of *cis-*/*trans*-interfacial residues in ECS2-ECH region participating in *cis-*/*trans*-interaction. SASA values [Å^2^] normalized to the side chain atom numbers mean of residues of participating subunits. (**G**–**J**) For pairs K69-E151 (**G**), R33-E155 (**H**), R33-E151 in trans (**I**), and R33-E151 in cis (**J**), number of pairs interacting in more than 35% of the time frames were counted and averaged for the four interfaces in the middle pore of dodecamer.

(i) For the face-to-face-*cis* interface type, the H-bonds between two β4-strands (F59 to P63 residue backbones) of subunit pairs of the middle pore were counted. The mean H-bond count per interface was ~ 1.7 for all models (Figure 3A) and thus in the same range as for CLDN10b and CLDN15 models (1–2, [15,27]). 

(ii) For the linear-*cis* interface, the distance between F68 (Cγ, ECH region) and L156 (Cγ, ECS2 pocket) was measured over time. For all CLDN10a models, the distance was constant over time, and for *IB-2*, *IB-2+lic*, *IB-2+hc*, *IB-2+ohc*~7.7 Å, and *IB-1*~6.6 Å (mean of all interfaces in dodecamer, Figure 3B). However, the mean distance differed between individual linear-*cis* interfaces within a dodecamer, as shown for *IB-2* as an example (Figure 3C). In addition, individual interfaces with at least one electrostatic interaction between the ECS2 pocket (E155) and ECH region (T66, I67, F68) were counted over time. For the different models, 4 ±1 out of 8 possible counts were obtained mostly and relatively constant over time (Figure 3D). Together, the measurements indicated that the linear-*cis* interface was largely maintained during the simulations for all CLDN10a model variants, similar to those shown for the corresponding CLDN10b model previously [15]. 

(iii) For the ECS2-ECS2-*trans* interface, the distance between the two P147 Cα atoms of two opposing subunits was measured over the simulation time. The distance was relatively constant with 6.4 ± 0.4 Å for *IB-1*, 7.4 ± 0.5 Å for *IB-2*, 7.5 ± 0.4 Å for *IB-2+lic*, 7.7 ± 0.3 Å for *IB-2+hc* and 8.3 ± 0.6 Å for *IB-2+ohc* models (Mean ± SD, Figure 3E). All were in a similar range as those obtained for CLDN10b (6.7–8.5 Å, [15]), indicating that ECS2-ECS2 proximity was maintained during the simulations. 

(iv) To evaluate the combined hydrophobic interactions mediated by the ECS2 (linear-*cis* with ECH region and *trans* with opposing ECS2), we calculated the solvent accessible surface area (SASA) of interfacial residues (measure for water exclusion at the interface, Figure 3F). SASA (normalized to the side chain atom numbers) was lowest for the hydrophobic pocket (F144, 156). Low SASA was also obtained for ECH region residues I67 and F68, whereas V70 had much higher SASA. *Trans*-interfacial P147, F149, and V150 showed intermediated SASA with the lowest values for P147. As comparisons, (a) the corresponding non-interacting residues (“free” in Figure 3F) in the peripheral subunits and (b) the close-by positively charged K69 (ECH region) and R33 (β1β2 loop) had much higher SASA. Comparing the different CLDN10a model variants, the more constrained models (*IB-2+lic*, *IB-2*-hc, and *IB-2*-ohc) had lower sum SASA than the weakly constrained models *IB-1* and *IB-2* (Figure 3F). The data indicate that I67, F68, F144, F145, P147, and L156 strongly contribute to hydrophobic *cis*-/*trans*- interaction in the ECS2 region.

(v) Finally, the CLDN10a-specific and positively charged K69 and R33 were analyzed concerning interaction with the close-by negatively charged E151 and E155. The number of residue pairs that interact in >35% of simulation time was counted. Out of eight possible counts, the following were detected: R33-E155 in the same subunit: 1–3 (0 for *IB-2+lic*); R33-E151 *cis*: 1–2 (0 for *IB-2*); R33-E151 *trans*: 1–4; K69-E151 *trans*: 1–3 (0 for *IB-1*). The data suggest that the pore-lining R33 and K69 residues considerably interact with negatively charged residues and thus contribute (i) to shielding these residues and improving anion attraction and (ii) to the stabilization of inter-subunit interactions (Figure 3G–J). 

#### 2.2.4. Definition and Comparison of Interfaces within Dodecamer by Residue Contact Maps: ECS1 Loop Clusters

**Figure 4:**
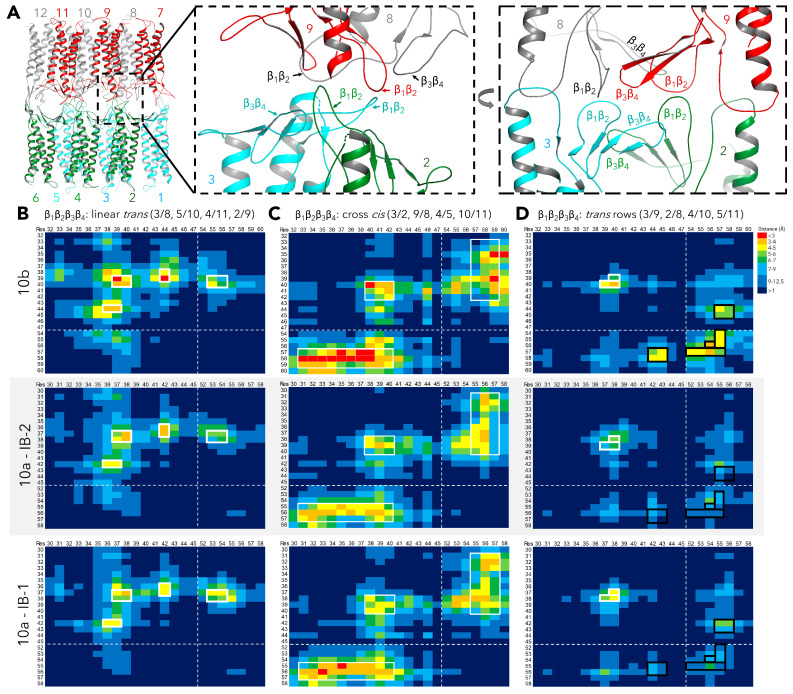
Contact maps for interfaces between different pairs of β1β2 and β3β4 loops in CLDN10b and CLDN10a models. (**A**) Overview. Left: Front view on CLDN10a dodecamer. Middle: A close-up of the dashed black box on the left shows a central cluster of different loops of neighboring subunits (different colors) in contact. Right: Turned view on this β1β2/β3β4 loop cluster showing another perspective on loop contacts. (**B**–**D**) Mean distances (closest atoms) between the numbered residues of β1β2 loops (CLDN10a: 30–45) and β3β4 loops (CLDN10a: 52–58) of three different subunit pairs. For the definition of pairs, see headlines and subunit numbering according to (**A**). The multiple individual interfaces in dodecamer were averaged. Residue contact pairs are boxed in white if similar, in red if shifted, and in black when different between CLDN10a and -10b. The contact maps provide interface fingerprints for comparison of the different claudin models. CLDN10b data taken from previous simulations [15].

The pore is in the central half on each side, bounded by *cis*- and *trans*-associations of β1β2– and β3β4 loops of four subunits (Figure 1G and Figure 4A, [15]). Here, three different pairwise subunit combinations (i–iii) come into contact (Figure 4A,B–D headlines). For each combination, respective residue contact maps were generated for the CLDN10a *IB-1* and *IB-2* simulations and compared with those of previous CLDN10b simulations [15]:

(i) Linear-*trans* pairs in a claudin row (Figure 4B): For CLDN10a/-10b residues in β1β2 loop, contact (<5 Å) of V37/V39, I38/I40 with V37/V39, I38/I40 and S36/T38, V37/V39 with W42/T44 and of V37/V39, I38/I40 with G53/T55, N54/D56 in β3β4 loop was detected. The pairwise distances were, on average, slightly higher for CLDN10a than for -10b.

(ii) Crosswise-*cis* pairs between claudin rows (Figure 4C): Contact of (CLDN10a/-10b residues) I38/I40, T39/T41, A40/T42 with I38/I40, T39/T41, A40/T42 in β1β2 loop and T31/S33 to A40/T42 with A55/S57 to G57/G59 in β3β4 loop. The overall contact pattern was similar for CLDN10a and -10b, though the distances were slightly larger for CLDN10a. In addition, the contact pattern shows some differences between CLDN10a-*IB-1*, -10a-*IB-2,* and CLDN10b models (see below Section 2.2.5). 

(iii) *Trans* pairs between claudin rows (Figure 4D): Also here, a similar overall pattern for both CLDN10 isoforms, but in sum, slightly larger distances for CLDN10a were obtained. I38/I40 contact with V37/V39, I38/I40 was similar for CLDN10a and -10b with increasing distances in the order CLDN10b, -10a-*IB-1*, -10a-*IB-2*. Distances for W42/T44, V43/Y45 of β1β2 loop with A55/S57, L56/T58 of β3β4 loop and A55/S57 with A52/V54 to N54/D56 and N54/D56 with N54/D56 of β3β4 loops were larger for CLDN10a than for 10b.

In total, the contact maps showed a similar overall pattern for CLDN10b and -10a models, reflecting a similar overall architecture of CLDN10a and- 10b channels. However, in sum, the pair distances are, on average, slightly higher for the CLDN10a dodecamer simulations.

#### 2.2.5. Further Analysis of β1β2 Loop Clusters

**Figure 5:**
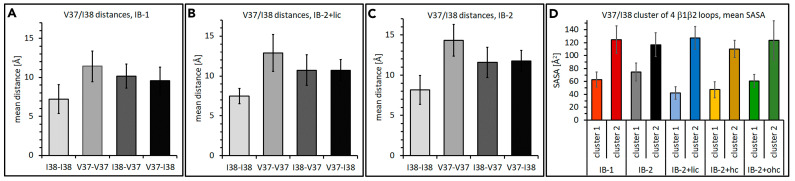
Analysis of hydrophobic β1β2 loop clusters in CLDN10a simulations. (**A**) IB-1 model. (**B**) IB-2+lic model (**C**). IB-2 model. Pairwise distances for V37-V37, I38-I38, V37-I48, V37-I48 (cis, Cβ-atoms). The last 50 ns of production runs were analyzed. Mean ± SD over time and four interfaces in dodecamer. (**D**) Solvent accessible surface area (SASA) for V37 and I38 residues of four interacting subunits, two clusters in each dodecamer model variant. Mean over time ± SD.

For CLDN10b, it was suggested that the β1β2 loop tips (conserved among classic claudins) of four subunits form a hydrophobic cluster [15]. Thus, the contribution of the β1β2 loop tip (V37, I38) of CLDN10a to hydrophobic cluster formation was analyzed. As mentioned above, the four loop tips can come into contact in three different combinations (Figure 4). As an example, for the crosswise-*cis* contact, the mean pairwise distances were calculated for V37-V37, I38-I38, V37-I38, I38-V37 (Cβ-atoms) and the different CLDN10a model variants (Figure 5A–C). The distances were between 7.2 and 14.3 Å. For all CLDN10a models, the I38-I38 distance was lowest (7.2–8.2 Å), and V37-V37 distances were highest (11.4–14.3 Å). The pairwise distances were similar to that of CLDN10b [15]. 

As a measure for hydrophobic clustering of the four loop tips, water exclusion was analyzed by SASA measurements. The mean SASA (during simulation time) for V37 and I38 of four interacting subunits were calculated for the two clusters in the dodecamer (left and right side of the middle pore, Figure 1G). For all models, the mean SASA differed considerably between the two clusters (42–74 Å^2^ and 110–127 Å^2^, Figure 5D). The lower value was similar to the values obtained for the corresponding CLDN10b models [15]. The higher value was still much lower than the SASA obtained for alternative CLDN10b models with a different β1β2 loop arrangement (195–411 Å^2^), [15]). However, the differing SASA for the two clusters of one dodecamer indicates an unexpected asymmetry. The latter might be due to the limited accuracy of the simulations. Thus, CLDN10a *IB-2,* which showed the smallest difference between the SASA of the two clusters, was chosen as the reference model.

In sum, the results indicate the formation of similar hydrophobic β1β2 loop clusters for CLDN10a and CLDN10b, supporting the contribution of these conserved interactions to oligomerization.

### 2.3. Ion Permeation Pathway of Pore in CLDN10a Dodecamer Models

**Figure 6:**
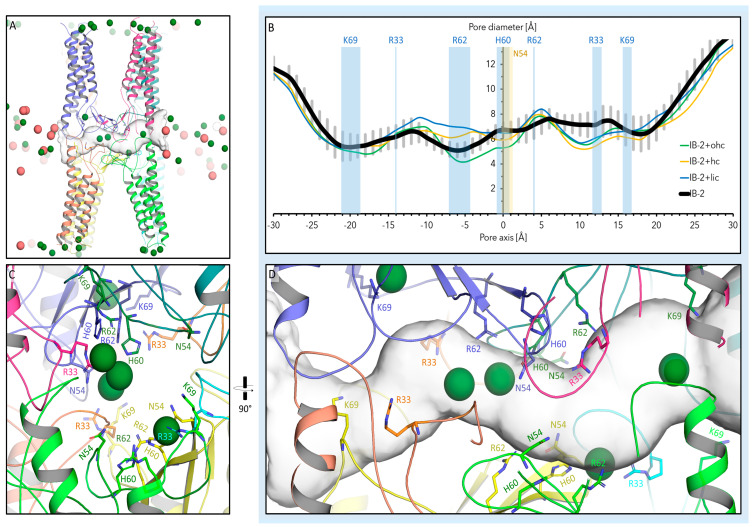
Ion permeation pathway of pore in CLDN10a models. (**A**,**C**,**D**) Snapshots at 100 ns for IB-2 model. Eight subunits lining the middle pore are shown as colored cartoons; the permeation pathway determined by the HOLE program is shown as a transparent gray surface, Na^+^ as red, and Cl^−^ as green spheres. (**A**) Overview: The pore axis runs from left to right. (**B**) Pore diameter profiles for CLDN10a IB-2, IB-2+lic, IB-2+hc and IB-2+ohc models. The pore profile of IB-2 is represented by a bold black line, with the corresponding SD values given as gray bars in addition. Mean diameter of last 50 ns of simulation along the pore axis. Pore pathway and diameter detection by HOLE. The position of the most relevant residues along the pore axis is indicated. (**C**,**D**) Close-up of permeation pathway in two different orientations. Most-relevant pore-lining residues are shown as sticks. R33 residues belong not to the tetrameric pore scaffold but to neighboring subunits. See also Video S2.

The ion permeation path in the CLDN10a dodecamer models (*IB-2*, *IB-2+lic*, *IB-2+hc,* and *IB-2+ohc*) was inspected by analyzing the diameter along the axis of the middle pore in the last 50 ns of the simulation (using HOLE). The pore profiles of the models differed up to 2.5 Å with respect to the mean diameter at particular positions along the pore axis (Figure 6B). The mean diameter differed also on both sides of the pore center that was surrounded by four N54 and four H60 residues (Figure 6B–D). For the reference model *IB-2*, the center showed a mean diameter of 6.7 Å. For *IB-2* and *IB-2+hc*, on one side, the R62 region formed the narrowest site (5.1 Å for *IB-2*), whereas on the other side, the R62 region was wider (7.4 Å for *IB-2*) than around H60. Nevertheless, for all models, the mean pore diameter in the inner pore region (spanning R33-R62-H60-R62-R33) was smaller than in the pore periphery beyond K69 residues. To what extent do the mentioned variations reflect the limited precision of the simulations in these details or the inherent flexibility of the CLDN10a channels? This will be a topic for further refinement studies. Nevertheless, the simulations indicate that the constriction site of the CLDN10a channels is formed in the central H60/R62 region. In sum, similar to CLDN10b channel simulations, those for CLDN10a showed constriction in the pore center with a similar minimal diameter of 5.1 Å (5.2 Å for CLDN10b *8IBno* [15]). This pore diameter is in agreement with experimental measurements [35,40]. While the pore center of CLDN10a and-10b channels were formed by the corresponding residues (N54/H60 in CLDN10a and D56/N62 in -10b), the constriction site for CLDN10a was not observed in the very center of the pore but next to it (R62). 

The most relevant residues lining the pore (~60 Å in length) are shown in (Figure 6C,D). The pore center is formed by four N54 and four H60 residues belonging to subunits of the tetrameric core barrel of the pore. In contrast, in the CLDN10b model, the pore center was formed by the D56 (negative) and N62 at the corresponding positions [15]. Next to these residues, the positively charged R62 and K64 are located in CLDN10a and- 10b, respectively. However, close to R62, another positively charged R33 is located in CLDN10a, whereas the negatively charged D36 is located close to K64 in CLDN10b [15]. Of note, R33/D36 residues are contributed by the interlocked β1β2 loop of neighboring subunits that are not part of the tetrameric core barrel (*octameric-interlocked-barrels* architecture [15]). In addition, further, in the direction of the pore periphery in the ECH region, K69 is present in CLDN10a instead of the non-polar A71 in CLDN10b. Both residues, K69 and R33, were capable of neutralizing at least transiently the negatively charged E151/E153 common for CLDN10a and -10b (Figure 3G–J). Furthermore, CLDN10a with Q45, N50, S58, H64, contains more additional polar residues than CLDN10b with the corresponding A47, A52, V60, F66 residues. With these mentioned and other residues (mostly identical for CLDN10a and -10b: K29/31, A71/D73 E143/145, D146/148, K139/141 (K153/K155, E155/157 mainly shielded), the pore has a net charge of +8 for CLDN10a and -12 for CLDN10b. Consequently, the CLDN10a pore was largely filled with Cl^−^ ions, whereas that of CLDN10b with Na^+^ ions (Figure 1 and Figure 6).

### 2.4. Interaction of Pore-Lining Residues with Ions in CLDN10a and CLDN10b Channels 

**Figure 7:**
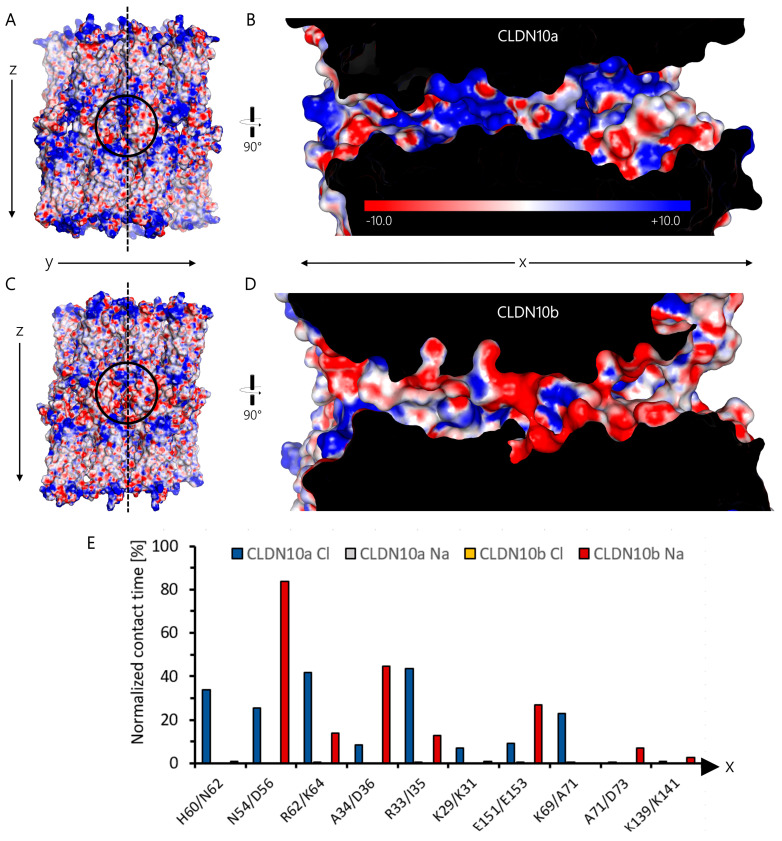
Electrostatic surface potential (ESP) map of CLDN10a (**A**,**B**) and CLDN10b (**C**,**D**) channel models. (**A**,**C**) Electrostatic potential mapped on dodecamer protein surface of 100 ns snapshots of CLDN10a *IB-2* and CLDN10b [15] simulations. Front view along y and z axes. (**B**,**D**) Clipped cross-section along x and z axes at the middle position of dodecamers (dashed line in (**A**,**C**)). The internal protein region is shown in black. Electrostatic surface potential calculated with Poisson–Boltzmann equation Solver (PBEQ-Solver) is shown color-coded (−10 to +10 kCal/mol.e). ESP of the pore-lining surface differs strongly between CLDN10a and -10b. (**E**) Contact time of relevant pore-lining residues with Cl^−^) and Na^+^ ions. The contact profile differs strongly between the two channels. The residues (CLDN10a/-10b) are ordered according to their position along the x-axis from the pore center towards the two-pore entrances. For the calculation of normalized contact time, the percentage of time frames in which the respective residue was closer than 4 Å to at least one ion was calculated for each subunit lining the central pore and averaged.

In order to compare the pore-lining surfaces of CLDN10a and CLDN10b channels, the electrostatic surface potential was calculated by solving the Poisson-Boltzmann (PB) equation using the PBEQ Solver (https://charmm-gui.org/?doc=input/pbeqsolver, URL accessed on 30 January 2024 [41]) (Figure 7A–D). Fitting to the above-mentioned opposite net charge of the two pores, the electrostatic potentials differed strongly: Mostly positive for CLDN10a but mostly negative for CLDN10b, especially in the inner half of the pore (Figure 7B,D). 

The pore-lining residues were further analyzed by measuring the contact time of relevant residues (see Section 2.3) of CLDN10a and CLDN10b with Na^+^ and Cl^−^ ions. In CLDN10b, the central D56 was predominantly in contact with Na^+^ ions (>80% of the time), D36 (>40% of the time), and E153 (>25% of the time) also showed frequent contact (Figure 7E). In contrast, contacts with Cl^−^ were very rare. In comparison, for CLDN10a, the opposite was obtained: Frequent contacts with Cl^−^ but very rare with Na^+^. However, for CLDN10a, no residue had a normalized contact time with Cl^−^ ions >45%, and the contacts were spread over more residues than for CLDN10b: The central H60 and N54 with ~34% and ~25% normalized contact time, respectively, and R62, R33, and K69 with ~42%, ~44% and ~23% normalized contact time, respectively. For both channels, charged residues in the inner part of the pore were frequently in contact with oppositely charged ions, whereas charge residues close to the entrance (K139 for CLDN10b, D73 for CLDN10a) had very infrequent contact with the ions (Figure 7E).

**Figure 8:**
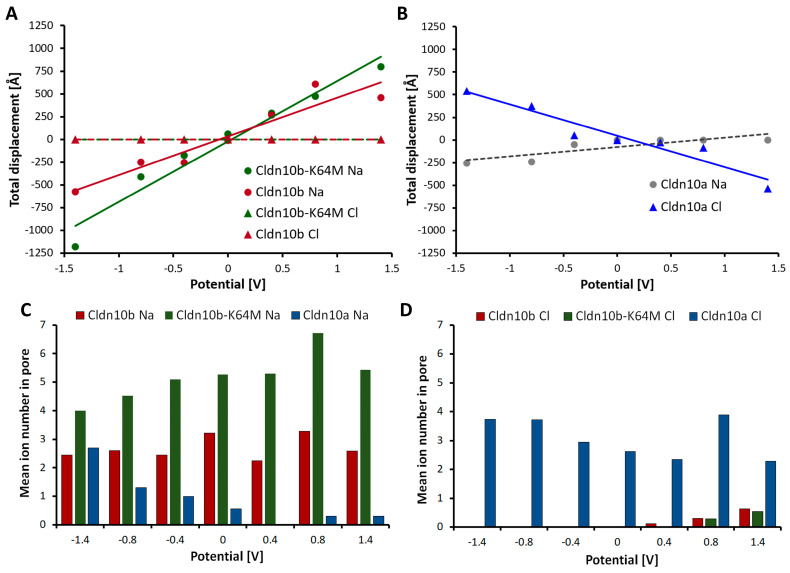
CLDN10a and -10b channel models show opposite charge selectivity. External electric fields were applied as driving forces, and total ion displacement was calculated over simulation time (50 ns). (**A**) For CLDN10b, the total displacement of Na^+^ (red dots) but not that of Cl^−^ (red triangles) depended linearly on voltage. For the CLDN10b-K64M mutant, the slope of the regression line was higher than for the wild type, suggesting slightly higher conductance for the mutant. (**B**) For CLDN10a, the absolute value of the slope of the regression line for Cl^−^ total displacement was higher than for Na^+^ total displacement, indicating the opposite charge selectivity of ion conduction compared to CLDN10b. In (**A**,**B**), the negative values of total displacement represent the movement in opposite directions on the same axis. (**C**) The mean number of Na^+^ ions in the pore for CLDN10b was much higher than for CLDN10a and highest for CLDN10b-K64M. (**D**) For CLDN10a, much more Cl^−^ ions were in the pore than for CLDN10b and CLDN10b-K64M. The mean number of Cl^−^ ions in the pore for CLDN10a was similar to the Na^+^ ion number for CLDN10b.

Finally, we investigated the charge selectivity of CLDN10 channels by applying an external voltage gradient (Figure 8). The low-resistance pathway on both sides of the dodecamer hindered measurement of the ionic current and calculation of the conductance as determined experimentally [35,39]. However, as a quantitative estimate of the ion movement through the channel and as an approximation of the charge selectivity of the pore, total ion displacement between the two ends of the channel over the simulation time was calculated. For CLDN10b, the total displacement of the Na^+^ ions depended linearly on the voltage and was between 578 Å in one direction for −1.4 V and 457 Å in the opposite direction for 1.4 V. In contrast, the total displacement of Cl^−^ ions was 0 Å for all voltages, clearly showing the cation selectivity of the channel (Figure 8A). We also analyzed the CLDN10b mutant K64M that was suggested to enhance Na^+^ ion interaction [15]. For CLDN10b-K64M, the slope of the linear fit of the relationship between the total ion displacement and voltage was slightly higher than for CLDN10b-wt, suggesting a slightly higher Na^+^ conductance for the mutant, as expected. For CLDN10a, the total ion displacement of Cl^−^ ions was 544 Å for −1.4 V and 536 Å for 1.4 V in opposite directions. The total ion displacement of Na^+^ ions was −255 Å for −1.4 V and 0 Å for 1.4 V. The absolute value of the slope of the linear fit for the Cl^−^ total displacement was higher than that for the Na^+^ total displacement, indicating the anion preference of the CLDN10a channel (Figure 8B). 

In addition, we determined the mean number of ions in the pore during the simulation time (Figure 8C,D). For CLDN10b much more Na^+^ ions were detected than for CLDN10a. For CLDN10b-K64M, even more Na^+^ ions were present. In contrast, many Cl^−^ ions were detected for CLDN10a but hardly any for CLDN10b and CLDN10b-K64M. Interestingly, the number of Cl^−^ ions for CLDN10a and that of Na^+^ ions in CLDN10b were in a similar range. No clear dependence of the mean ion number and the voltage gradient was observed for CLDN10b and Na^+^ as well as CLDN10a and Cl^−^. However, with decreasing voltage, the Na^+^ ion number increased for CLDN10a, decreased for CLDN10b-K64M, and the Cl^−^ ion number increased slightly with increasing voltage for CLDN10b and CLDN10b-K64M. These voltage dependencies might reflect model asymmetries that are enhanced by strong voltage gradients. However, in sum, the data obtained with the electric field simulations demonstrated opposite charge-dependence of attraction and conduction of ions for the CLDN10a and -10b channel models, respectively. 

## 3. Discussion

In this study, we investigated paracellular anion channels formed by CLDN10a using homology modeling and MD simulations. We compared the channels with those formed by CLDN10b simulated previously [15] with respect to interfaces between subunits, pore-lining residues, and ion passage. The results indicate that CLDN10a and -10b channels share a common architecture: Interlocked pore barrels as part of a joined double (=quadruple) rows arrangement (*JDR*) of claudin subunits within TJ strands. In addition to conserved interaction motifs, some inter-subunit interfaces differ between the two isoforms. Furthermore, pore-lining residues differing between CLDN10a and -10b channels cause opposite charge selectivity. The simulations are very well in line with experimental data and provide, for the first time, information on a complete and prototypic paracellular anion channel on the atomic level.

Tracer flux assays and electrophysiological measurements such as transepithelial resistance (TER) or dilution potentials of cellular monolayers are essential to characterize the functionality of TJs constituted by claudins. In combination with mutagenesis and biochemical studies, they can be used to identify sequence determinants for the formation of TJ strands, barriers, and channels [2,42]. However, these methods provide only limited information about the structure and molecular dynamics of claudin polymers, TJ strand formation, and how pore-lining residues aid in the ion permeation process. Interestingly, boosted by the first claudin crystal structure solved by Suzuki et al. [9,10], computational modeling and MD simulations have contributed significantly to the understanding of claudin oligomerization and pore formation. 

Although different molecular models of claudin-based TJ strands and ion channels have been reported and refined, their validity and differences between the claudin subtypes are disputed [4,12,15,18,19,20,21,22,23,25,26,27,28,29,43,44]. This is also due to the fact that in contrast to transmembrane channels and other cell-junctional structures, no experimental structure has been resolved for TJ-like claudin oligomers so far [4] (https://www.rcsb.org/ URL accessed on 10 January 2024). Comparison of the channel-forming CLDN15, CLDN10b, and CLDN10a in this and a previous study [15] by MD simulations support the idea that at least these three claudins form channels and strands according to the *JDR* arrangement suggested by Suzuki et al. [10]. The linear-*cis* and face-to-face-*cis* interfaces of this arrangement model are supported by several experimental structure-functions studies on CLDN15, -10b, -5, -3, -2 and -1 [8,10,11,12,19,24,45,46]. Regarding the *trans*-interfaces, critical contributions by the β1β2 loop of ECS1 and the ECS2 have been proposed, but the interaction patterns were largely unclear [8,10]. Modeling and MD simulation studies suggested two different conformational variants by which the β1β2 loop participates in *cis*-/*trans*-oligomerization of classic claudins and in conjunction of adjacent tetrameric subunit repeats within strands. In the first variant, the β1β2 loop shows a rather straight extension and orientation towards a subunit in the opposing membrane. This results in a *tetrameric-locked-barrel* (*4LB*) that forms a pore in the case of channel-forming claudins and is nearly completely lined by residues from only four subunits [12,15]. To a certain extent, similar tetrameric pore variants have been reported earlier [25,28]. In the second variant, the β1β2 loop is oriented flatter towards a subunit in the same membrane (Figure 1D). This results in *interlocked-barrels* (*IB*) and pores (in the case of channel-forming claudins) that are lined, especially in the inner region, by eight subunits [15,26,27] (Figure 1C,G). Comparison of both variants for CLDN10b and CLDN15 by MD simulations supported the *IB* model [15]. 

Consequently, in this study, we simulated *IB* models for the comparison of CLDN10a and CLDN10b channels. Key results for CLDN10a model variant *IB-2*, which was chosen as the reference model, and the previously generated CLDN10b *IB* model [15] are summarized in Table 1. The equilibrated CLDN10a dodecamers showed an overall architecture similar to that of CLDN10b. This was expected since a CLDN10b template was used to generate the CLDN10a homology dodecamer models. More importantly, the CLDN10a model variants showed similar stability as the CLDN10b models: RMSD of dodecamer protein backbone was similar (Figure S4), contact map pattern (Figure 2 and Figure 4), as well as interaction parameter for face-to-face, linear-cis, ECS2-ECS2-trans (Figure 3) and hydrophobic cluster of β1β2 loop tips (Figure 5), were in most cases slightly higher for CLDN10a models than for CLDN10b models [15], however still in similar ranges. All interfaces and an open pore conformation were preserved during the simulations (Table 1). Thus, the results support the *IB* model for CLDN10a and suggest that at least CLDN15, -10b, and -10a share a similar overall architecture: Adjacent, sidewise-unsealed tetrameric pore scaffolds that are interlocked via β1β2 loops. These loops mediate, together with ECS2, *cis*- and *trans*-interaction between subunits of adjacent tetramers. The clustering of the hydrophobic β1β2 loop tips of four subunits was proposed to be a common driving force for the oligomerization of classic claudins for which the hydrophobicity is conserved [4,7,13]. 

The linear-*cis* interface consisted of hydrophobic and electrostatic interactions for both CLDN10a and -10b. However, of note, while the participating ECH region of CLDN10b showed an α-helical conformation similar to the crystal structure and oligomer models of CLDN15 ([9,10,15,25,26,27], the corresponding region of CLDN10a showed mainly a non-α-helical conformation (Figure 2A, right), similar to other claudin crystal structures [4] and claudin AlphaFold models. Interestingly, conformational differences in the ECH region and ECS2 pocket (together mediating the linear-*cis* interaction) were suggested to influence CLDN3 oligomerization and strand bending [11]. In addition, the sliding of CLDN15 subunits relative to each other close to the linear-*cis* interface was suggested to influence strand flexibility [27]. Thus, our CLDN10a data further support the idea that the conformation of regions participating in the linear-*cis* interaction can vary over time or between claudin subtypes or junctional conditions.

We observed that lipids were trapped in the central region between the two claudin rows of one membrane (Figure 1B,F). In the corresponding protein region of two experimental claudin structures (PDB ID: 4p79 [9], 8U4V [47]), lipid-mimicking detergent molecules were resolved and thus stably associated with the claudins. This experimental finding and the uncommon protein-lipid arrangement in the model are in line with previous studies that suggested claudin polymers in TJs to be associated with a special lipid environment [2,48,49,50]. 

Strikingly, the ion conduction pathway differed strongly between CLDN10a and -10b in the inner half of the pore (Figure 1 and Figure 6). While the pore center in CLDN10a models is formed by N54 and H60 residues, it is formed by D56 and N62 residues at the corresponding positions in CLDN10b models. Close to the center, both claudins contain a different positively charged residue (R62/K64). Next to this position, CLDN10a contains another positively charged R33, whereas in CLDN10b, the negatively charged D36 is located. Importantly, these residues are contributed by the interlocked β1β2 loop of adjacent subunits that are not part of the tetrameric core barrel. Further to the pore periphery, the charged K69 is present in CLDN10a instead of the non-polar A71 in CLDN10b. CLDN10a also contains more additional polar pore-lining residues than CLDN10b. In sum, this leads to an opposite electrostatic potential on the pore-lining surface for the two different claudin channels (Figure 7A–D). Consequently, the pore-lining residues of CLDN10a attracted and interacted strongly with Cl^−^ ions, whereas that of CLDN10b did so with Na^+^ ions (Figure 7E and Figure 8C,D). This resulted in anion transport through CLDN10a channels in contrast to cation transport through CLDN10b channels (Figure 8). Thus, in total, the simulations fit very well to the charge-selectivity of the two claudin channels and contributions of ECS1 residues, in particular of R33 and R62 in CLDN10a, that were demonstrated by electrophysiological measurements [35,36,38,39]. 

As mentioned above, the equilibrated CLDN10a and CLDN10b models were further used to study the ion permeation. However, it is important to mention that reproducing the physiological ion transport through claudin channels using computational models is complicated due to various possible sources of error [51]. During an all-atom MD simulation of a claudin pore model, movement of only a few ions through the pore could be observed because of the limited simulation time. Moreover, since these paracellular pores do not run through a membrane barrier, it is very challenging to calculate a net flux in such simulations. Different previous computational studies on claudin channel models approached this problem in different ways. Alberini et al. [43] and Irudayanathan et al. [18,52] observed the traverse of different ions through claudin channel models by calculating the potential of mean force (PMF) profiles using Umbrella sampling simulations. Samanta et al. [26] calculated the total ionic currents of cations through CLDN15 channels after applying a constant electric field corresponding to a transepithelial potential during the simulation. We followed a similar methodology in this study; however, there are some important limitations. One important issue is the use of conventional periodic boundary conditions (PBC), which results in a continuous bulk solution; hence, the ions are free to diffuse through boundaries. Samanta et al. countered this problem by allowing the claudin strands to continue across the PBCs, as a non-continuous strand (as in our system setups) will result in a low-resistance pathway on the two ends of the periodic box, which results in leakage of ions. To address this, we concentrated only on the ion movement through the middle pore and not the whole system by considering an imaginary cuboid through the pore. The ion movement events occurring in the cuboid were observed and quantified using displacement calculations. Although this approach hinders the calculation of current and conductance through the channels across an epithelium as measured experimentally [35,39], it allows a relative comparison of ion movements through the different claudin channels. 

In total, this MD simulation study provides the first atomic model of a complete and prototypic paracellular anion channel and suggests that CLDN10a anion and -10b cation channels share the same overall architecture but differ mainly in their pore-lining residues. This is of high interest since nearly all transcellular anion channels differ strongly from cation channels in their architecture [53]. However, one has to keep in mind that claudin-based paracellular channels differ strongly in structure from transmembrane channels since the ion pore runs parallel to instead of through the membrane. A similar overall architecture for paracellular anion and cation channels also fits the fact that both are—in contrast to transmembrane channels—embedded in protein polymers that form a functional barrier against larger solutes. 

Certainly, the first structural model reported here cannot reflect all structural and mechanistic details of native CLDN10a channels. On the one hand, more structure-function studies employing mutagenesis and electrophysiology have to be performed to identify more sequence determinants for defined channel properties. On the other hand, more detailed MD simulations that take the in vitro data into account have to be performed. Varying details of regional conformations in the starting models, the lipid composition of the membrane, ions, force fields, release of remaining constraint (C61), number of subunits, and longer simulation times can be used for structural refinement in further studies. Thereby, for instance, it is expected to reduce channel asymmetries and, in turn, increase the accuracy of the simulations. In addition, free energy calculation can be used to analyze selectivity determinants in more detail [18,21,23,43]. Furthermore, progress in claudin complex structures resolved by cryo-electron microscopy [53] is expected to provide additional substantial insights into the structure of claudin channels and strands.

In sum, we generated a structural model for the prototypic anion-selective CLDN10a channel. Molecular dynamics simulations of CLDN10a or CLDN10b dodecamers indicate that both CLDN10 isoforms share the same channel and strand architecture: Sidewise unsealed tetrameric pore scaffolds, interlocked with adjacent pores via β1β2 loop of ECS1, leading to strands with claudin subunits arranged in double rows in one and in four joined rows in two membranes. Many interaction modes are suggested to be conserved among CLDN10a, -10b, and also -15. However, pore-lining residues differing between CLDN10a/-10b (i.e., R33/I35, A34/D36, K69/A71, N54/D56, H60/N62, R62/K64) result in opposite charge selectivity of the two channels. The findings are consistent with electrophysiological studies and thus provide novel mechanistic information about epithelial transport.

## 4. Materials and Methods

### 4.1. Modeling and Simulation Platform

Claudin 10a dodecamer model variants were modeled and simulated using the Schrödinger Maestro BioLuminate software (BioLuminate, version 4.9.134, Release 2022-4, Schrödinger, LLC, Mannheim, Germany, 2022) in Linux-x86_64 platform. Representation of the models in figures was generated using BioLuminate and Schrödinger PyMOL 2.5.2 (http://www.pymol.org/pymol URL accessed on 24 January 2022). 

### 4.2. Modeling of CLDN10a IB Dodecamer Model

A homology model of human CLDN10a (Uniprot P78369-2, M1-N184) was developed using the ‘Build Homology model’ module in Maestro BioLuminate. A well-equilibrated CLDN10b dodecamer subunit [15] was used as the modeling template. In addition, the conformation of the ECH region (66–71) was grafted from a CLDN10a model predicted using ColabFold (https://colab.research.google.com/github/sokrypton/ColabFold/blob/main/AlphaFold2.ipynb, URL accessed on 5 April 2023) [54,55]. An initial CLDN10a dodecamer model was modeled similarly to the previously published CLDN10b dodecamer models by replicating the CLDN10a homology model and aligning it with the CLDN10b dodecamer [15]. The CLDN10a dodecamer model consisted of two *trans*-interacting hexamers, resulting in a triple-pore model. A series of minimizations based on different experimental- and hypothesis-derived distance constraints [4,12,15,38,39] were performed using the ‘Macromodel minimization’ tool available in Maestro BioLuminate. The Polak–Ribiere Conjugate Gradient (PRCG) method that uses the Polak–Ribiere first derivative method was used [56]. The RMS gradient of the energy with respect to the coordinates in kJ mol^−1^ Å^−1^ was selected as the convergence criterion. After minimization, a series of molecular dynamics (MD) simulations in a water-solvated environment was followed to pre-equilibrate the model, considering the same distance constraints. The following considerations based on conservation of (a) sequence motifs and (b) strand formation among classic claudins [4,12,15,38,39] were followed during model equilibration:

i. Hydrophobic interface formed by the tip of β1β2 loops (V37 and I38)
ii. Face-to-face (*ftf*) interface (C61-C61 H-bonds)
iii. Linear-*cis* interface, where F68 (ECH) resides inside the pocket formed by F144, F145 and L156 (ECS2), and
iv. Hydrophilic interaction between E155 (ECS2) and the backbone or side chain of ECH region residues T66 and I67 are formed.
v. The CLDN10a-specific, positively charged residues R33 and K69 are oriented towards the anion-selective pore.

### 4.3. MD Simulations of CLDN10a IB Dodecamer Models

‘Desmond Molecular Dynamics’ module (Desmond Molecular Dynamics System, D. E. Shaw Research, New York, NY, USA, 2022. Maestro-Desmond Interoperability Tools, Schrödinger, New York, NY, USA, 2022; [15,57]) present in Maestro BioLuminate was used to perform the MD simulations of the CLDN10a dodecamer. The pre-equilibrated CLDN10a *IB* dodecamer model was embedded in two 1-palmitoyl-2-oleoyl-sn-glycero-3-phosphocholine (POPC) lipid bilayers. An equilibrated membrane from the CLDN10b *8IB* (short: *IB*) model [15] was used to follow a similar positioning of the dodecamer in the membranes. The membrane was further refined to remove lipids clashing with protein and to add missing lipids to avoid water inclusion. The double membrane-dodecamer complex was put into a simulation box of size 165, 165, and 150 Å in the x-, y- and z-axis, respectively. The orthorhombic box was solvated with TIP3P water molecules [58], physiological salt of 0.15 M Na^+^Cl^−,^ and charge-neutralizing Cl^−^ ions. OPLS4 force field [59] was applied during the simulations performed in the NPT ensemble at 310 K and 1.01325 bar. Nosé–Hoover chain method [60] and Martyna-Tobias-Klein method [61] were used as thermostats and as barostats, respectively. The short-range cutoff method with a cutoff radius of 9.0 Å was used to calculate Coulombic interactions. Bonded interactions were integrated using the RESPA algorithm at 2.0 fs timestep. The system was first relaxed into a local energy minimum through a ‘Desmond minimization’ available in Maestro BioLuminate, using a Brownian motion simulation run for 100 ps, followed by the NPT BioLuminate default relaxation protocol with added constraints on protein except for H atoms. Subsequently, a series of equilibration steps were performed for 160 ns by gradually releasing the constraints. In the beginning, a force constant of 10 kcal mol^−1^ Å^−2^ was applied to the whole protein. Afterward, first side chains, then backbone atoms, stepwise for loops, β-sheets, and helices were gradually released over time by decreasing the respective force constants stepwise from 5 to 0 kcal mol^−1^ Å^−2^. 

Finally, only the backbone atoms of C61 (cysteine 61) were constrained with 1 kcal mol^−1^Å^−2^ to perform a first 100 ns production run for analysis (i. *IB-1, Interlocked Barrels model 1*). These weak constraints were kept as compensation for simulation parameters that might not fully reflect the native environment (e.g., the unknown TJ lipid composition) and to support the previously suggested [10,12,15,25,26,27,46] face-to-face (ftf) interface that includes C61-C61 H-bonding. 

Additionally, one of the predecessors of the above-mentioned CLDN10a *IB-1* model was refined further to increase the interface-wise symmetry of the model by adjusting some interfacial residues (such as S35, S36, L56, F68, P147, Q152) using ‘Maestro builder’. The refined model *IB-2* was embedded in a double membrane, minimized, stepwise equilibrated, and simulated similarly to the *IB-1* model. For analysis of the *IB-2* model, four variant production runs were performed that differed in the following constraints:

i. *IB-2*—same constraints as *IB-1:* Force constant of 1 kcal mol^−1^ Å^−2^ on C61 backbone, all other atoms free.
ii. *IB-2+lic*– additional 1 kcal mol^−1^ Å^−2^ on backbone atoms and β-carbon of I67 and F68 to weakly constrain linear *cis*-interface (*lic*),
iii. *IB-2+hc*—additional 1 kcal mol^−1^ Å^−2^ on backbone atoms of helices to constrain mainly the transmembrane segments,
iv. *IB-2+ohc*—additional 1 kcal mol^−1^ Å^−2^ on backbone atoms of helices of the outer chains, i.e., chains that are not part of the middle pore.

Modeling and simulation steps that lead to the above-mentioned model variants are summarized in Figure S3. 

### 4.4. Analysis of the MD Trajectories

We extracted relevant data, including RMSD, interactions like hydrogen bonds and salt bridges, and distance between residues from simulation trajectories using the internal tools available in BioLuminate. The extracted data were compiled and analyzed using Microsoft excel (Microsoft Corporation, 2018, Microsoft Excel, Redmond, WA, USA). The analysis of Solvent-Accessible Surface Area (SASA) was performed using Visual Molecular Dynamics [62] (VMD, version 1.9.4a55, release 2021-10, https://www.ks.uiuc.edu/Research/vmd, IL, USA). 

MDAnalysis (version 2.3.0) [63,64] scripts written using Python3 [65] were developed to perform the analysis and visualization of pore dimension using the HOLE program [66,67] and residue-residue interaction analysis using contact maps. More information on the scripts can be found in our previous publication [15]. 

### 4.5. Applied Electric Field Simulations

We performed simulations by applying a potential bias in the axis parallel to the membrane surface and through the paracellular pores. One of the well-equilibrated structures from the production runs of CLDN10a (*IB-2*) and CLDN10b (*8IBli*) [15] simulations was selected. The protein dodecamer structures, along with the membranes, ions, and water, were converted into a new simulation system, energy minimized, and then equilibrated for 5 ns. During equilibration, the protein and the head group of lipid molecules were constrained with a force constant of 1 kcal mol^−1^ Å^−2^. Using the ‘e_bias’ plugin available in the Desmond simulation platform, simulations with applied electric fields along the membrane surface with different potentials like 1.4 V, 0.8 V, 0.4 V, −0.4 V, −0.8 V, and −1.4 V were simulated. During the simulations, the extracellular segments were kept free, and only the backbone atoms of the transmembrane helices were constrained with a force constant of 1 kcal mol^−1^ Å^−2^ to avoid their movement in the direction of the current due to the sudden application of the potential. The production runs were simulated for 50 ns in the NVT ensemble.

### 4.6. Calculation of Ion Total Displacement 

As a quantitative measure for the ion permeation, using MDAnalysis, the total displacement of Na^+^ and Cl^−^ ions through the pore was calculated using the coordinate information from the trajectories. The displacement calculation was performed for only the ions that cross a bottleneck in the center of the middle pores of CLDN10a and CLDN10b dodecamers. For the bottlenecks, the tetrads of H60 in CLDN10a and D56 in CLDN10b were considered, respectively, as these residues from four different chains come together at the center of the pore pathway. So, any ion that passes the center of mass of these four residues was considered to be passing the pore. However, a complication in choosing the ions traveling and passing only through the pore and not through any other side arose. For this purpose, we set boundaries in x, y, and z-axes to consider an imaginary cuboid through the pore, and only the ions that pass the pore within the cuboid were selected for the displacement calculation. The Cartesian coordinates of the C_α_ atom of K139 (K141 in CLDN10b) from the four chains that are located at the entrance of the middle pore on either side were used as the boundaries of the cuboid (Figure S6). The displacements of the filtered ions were calculated by extracting the position information of these ions along the axis parallel to the pore. The calculation of the total displacement (*D*) was derived from the following formula:

(1) $$D=\sum_{i=1}^{N}(x^{i}\left(t+1\right)-\left(x^{i}\left(t\right)\right)$$

where *N* is the number of ions, *x^i^* is the x-coordinate of an ion *i* at time *t*. 

Additionally, the mean number of Na^+^ and Cl^−^ ions in the cuboid over time was calculated for each simulation.

## Figures and Tables

**Table 1:** Comparison of key parameters of CLDN10a and CLDN10b channel models.

Parameter	CLDN10a (*IB-2*)	CLDN10b
Architecture	interlocked pore barrels, JDR; (Figure 1)	interlocked pore barrels, JDR; (Figure 1) [15]
RMSD (dodecamer)	~1.7 Å; (Figure S4A)	1.5–2.2 Å; Figure S4A
Linear-cis interface	maintained, non α-helical ECH region, F68 in, I67 close to ECS2 pocket; (Figure 2 and Figure 3)	maintained, α-helical ECH region, M69 but not L70 in ECS2 pocket
Face-to-face interface	maintained; (Figure 2 and Figure 3)	maintained; [15]
ECS2-ECS2-trans interface	contact maintained, flexible; (Figure 3),F144, F145 close to P147; (Figure 2)	contact maintained, flexible; [15]; F146, F147 close to P149, P149 close to P149; (Figure 2), [15]
β1β2loop tip cluster: V37/39,I39/I40 proximities	*trans* < 4 Å, *cis* < 5 Å; (Figure 4)	*trans* <3 Å, *cis* <3 Å; (Figure 4), [15]
β1β2loop tip cluster: V37/39,I39/I40 SASA	74- 117 Å^2^; (Figure 5)	49-70 Å^2^; [15]
Minimal pore diameter	~5.1 Å; (Figure 6)	~5.2 Å; [15]
Pore center lined by **Bold:** frequent ion contact	4× **N54**, 4× **H60** (Ø ~6.7 Å); (Figure 6)	4× **D56**, 4× N62 (also narrowest site); [15]
Charged residues (pore center to periphery) **Bold:** frequent ion contact	**R62**, **R33**, (E155), **K69**, E151, K29, E143, D146, K139; (Figure 6)	K64, **D36**, (E157), **E153**, K31, E145, D148, D73, K141; [15]
Other pore-lining residues	Polar: Q45, N50, S58, H64	Nonpolar: A47, A52, V60, F66
Pore net charge	+8	−12
Electrostatic surface potential of pore	mainly positive; (Figure 7)	mainly negative; (Figure 7)
Charge selectivity of channel	Strong anion attraction, anion conductance; (Figure 7 and Figure 8)	Strong cation attraction, cation conductance; (Figure 7 and Figure 8)

## Data Availability

The data presented in this study are available within the article and the Supplementary Materials. Further inquiries can be directed to the corresponding author.

## Supplementary Materials

The following supporting information can be downloaded at https://www.mdpi.com/article/10.3390/ijms25063161/s1.
