# Supplementary material for: Molecular Dynamics Simulations of Claudin-10a and -10b Ion Channels: With Similar Architecture, Different Pore Linings Determine the Opposite Charge Selectivity
Source: Int J Mol Sci. 2024 Mar 9;25(6):3161. doi: 10.3390/ijms25063161 (PMC10970694; doi:10.3390/ijms25063161)
Supplement: Supplementary file 1 [file ijms-25-03161-s001.zip › Supplemental files/ijms-2845628-supplementary material.pdf]

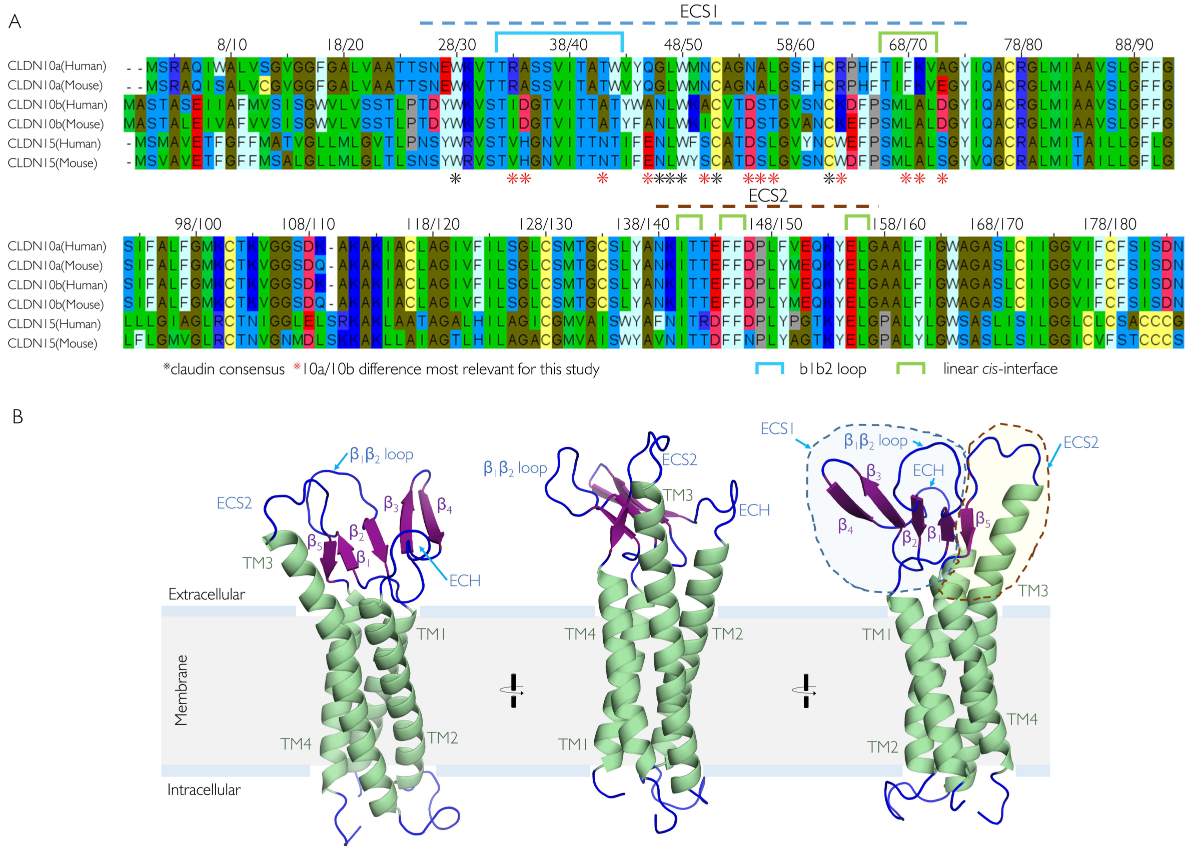

Figure S1. **(A)** Sequence alignment of human and mouse CLDN10a, -10b and CLDN15. N-terminus to end of TM4. Extracellular segements (ECS1 and ECS2), residues belonging to the β1β2 loop, linear-*cis* interface, claudin consensus sequence and residues differing between CLDN10a and -10b relevant for this study are marked. Numbering acording to CLDN10a/-10b. Residue color: Negative, red; positive, dark blue; polar, blue; small, olive; hydrophobic, green; aromatic, light cyan; cysteine, yellow; proline, gray. **(B)** CLDN10a homology model with marked structural elements: TM1 to 4: Transmembrane helices; β-sheet with β1 to β4 strands from ECS1 and β5 strand from ECS2; ECH region of ECS1: Region between β4 strand and TM 2 with varying extent of α-helical conformation; ECS2 consisting of extracellular extention of TM3, ECS2 loop/turn and β5 strand. Model based on a subunit of the CLDN10a dodecamer model *IB-1*.

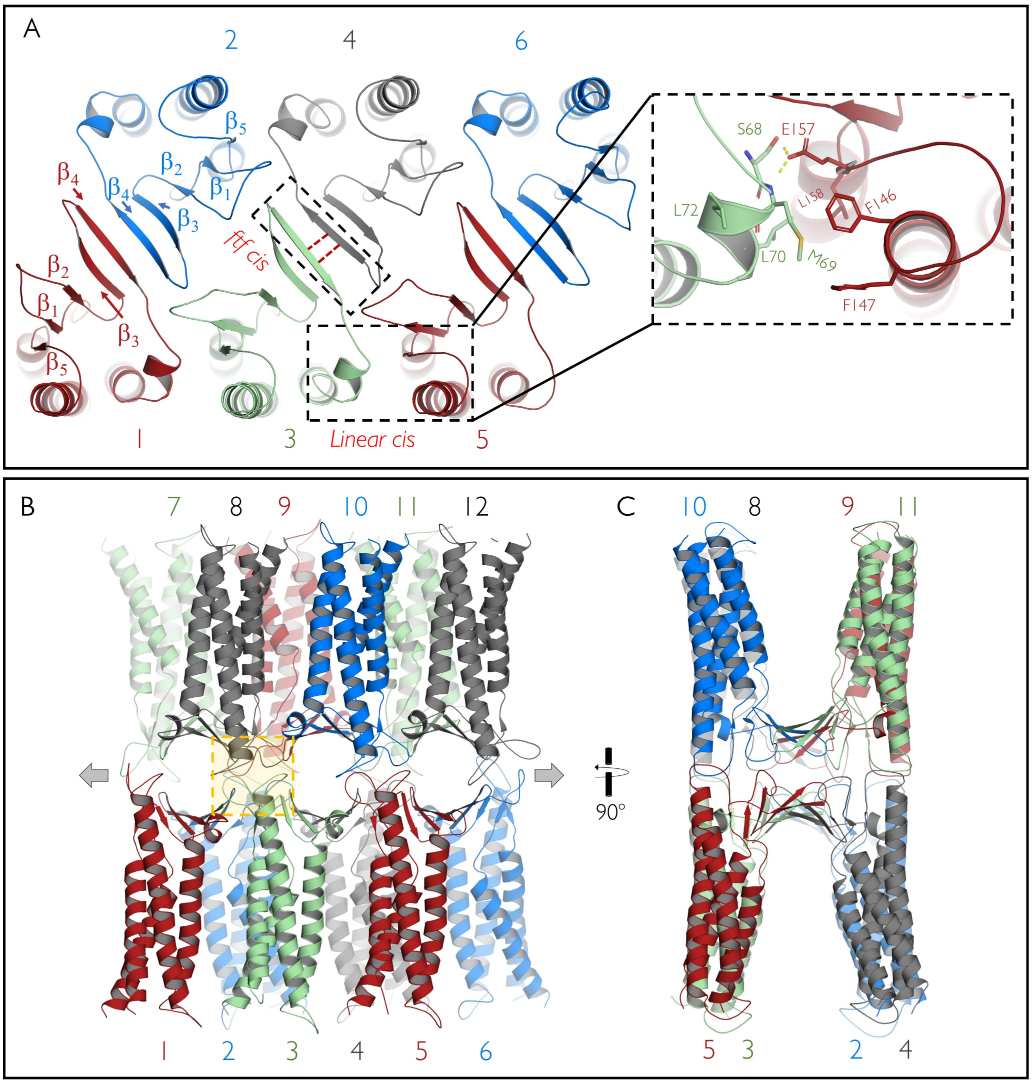

Figure S2. CLDN10b dodecamer model highlithing *cis*- and *trans*-interactions contributing to claudin polymerization. **(A)** In one membrane, multiple claudin subunits form an anti-parallel double row by linear-*cis* and face-to-face(ftf)-*cis* interfaces (dashed boxes). Top view from extracellular side, six subunits (1–6) are shown, β-strands 1–5 and H-bonds (red dashed lines) between β4-strands are marked. Close-up shows details of linear-*cis* interface: Hydrophobic M69 of extracellular helix (ECH) region sticks into hydrophobic pocket formed mainly by F146, F147 and L158 of extracellular segment two (ECS2); electrostatic interaction between E157 (ECS2) and S68 and ECH backbone. **(B)** Side view on 12 claudin subunits (1–12). Joined (from two membranes) double rows (JDR) architecture comprising extracellular *cis*- and *trans*-interfaces (latter: yellow dashed box) resulting in β-barrel-like pore scaffold. Arrows indicate strand elongation by additional subunits. **(C)** Turned-view on central pore lined by eight subunits. Based on CLDN10b model (8IBli, [1]).

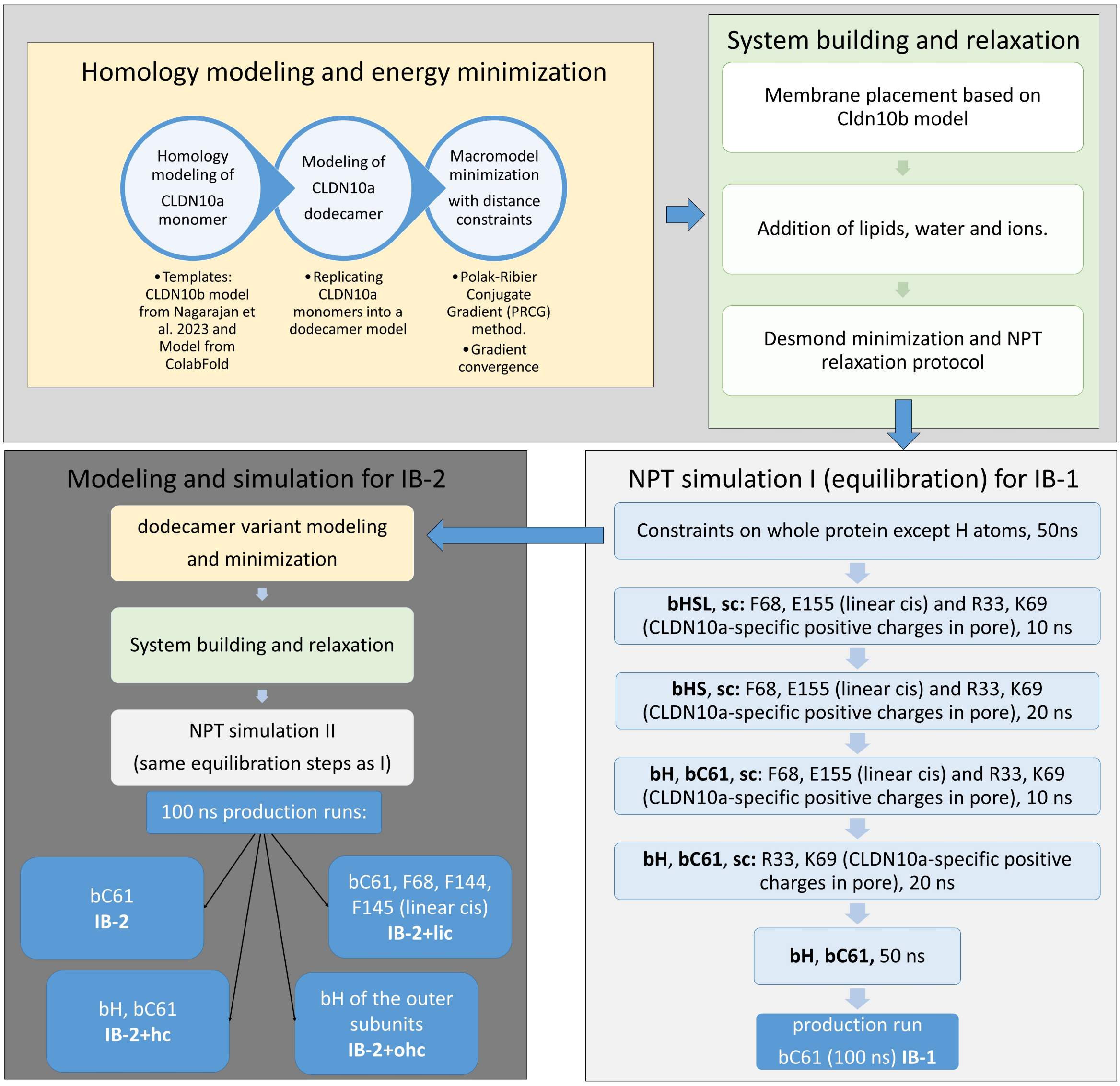

Constraints on: b – backbone atoms, H – Helices, S – Strands, L – Loops, sc – Side chains

Figure S3. Flowchart summarizing the modeling and simulation steps that lead to the different model variants. Simulations were performed in NPT ensemble (constant particle Numbers, Pressure, Temperature). For NPT simulation – I and – II, the constraints included in each steps are mentioned. In NPT simulation-II, all the steps mentioned in NPT simulation-I are followed until bH, bC61 (50 ns), and the subsequent differences in the constraints lead to the IB-2 variants. During the equilibrations steps the force constants were stepwise reduced from 10, 5 or 3 to 1 kcal mol^{-1} Å^{-2}. Force constant for bC61 was 1 kcal mol^{-1} Å^{-2}. All non-mentioned atoms were fully free to move during the simulations.

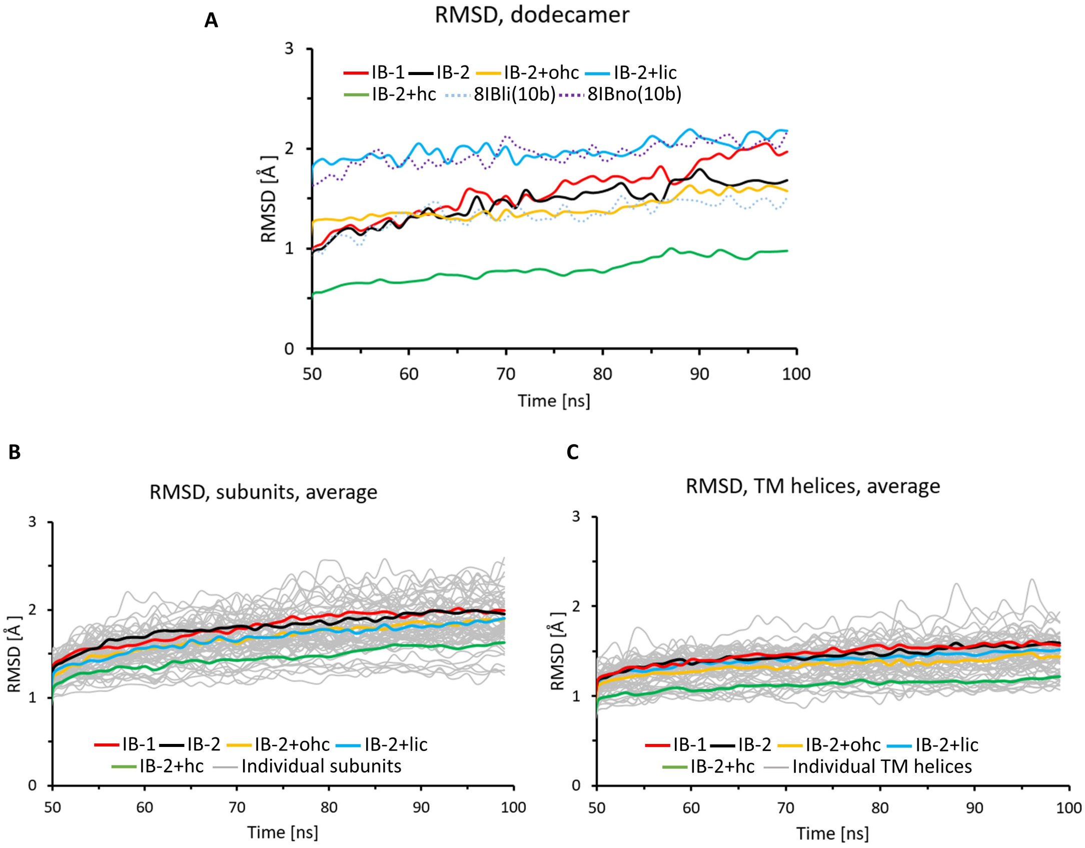

Figure S4. Root-mean-square deviation (RMSD) of protein backbone for CLDN10 dodecamer IB model variants, plotted over the last 50 ns of the production run simulation. CLDN10: IB-1, IB-2, IB-2+ohc, IB-2+lic, IB-2+hc; CLDN10b: 8IBli, 8IBno [1]. **(A)** RMSD for the entire dodecamer with respect to its initial structure (0 ns). Whereas RMSD of IB-1 increased steadily, it reached a plateau for all IB-2 CLDN10a and the two CLDN10b models at least after ~ 90 ns. It was lowest (~1.0 Å) for the strongest constrained one (IB-2+hc) and highest (~2.2 Å) for the run with constraints on the linear-cis interface (IB-2+lic). For IB-2 and IB-2+ohc, it showed an intermediate RMSD of ~1.7 Å. **(B)** RMSD for each subunit of the CLDN10a dodecamer with respect to its initial structure (0 ns). For each of the dodecamer subunits of the 5 different CLDN10a model variants (in total 60 subunits) RMSD is shown in light gray to reflect the heterogeneity between individual subunits. Mean RMSD of the 12 subunits of each dodecamer model variant is shown in bold. The values were relatively constant after ~ 70 ns indicating convergence. The mean RMSD was similar for *IB-1*, *IB-2*, *IB-2+lic* and *IB-2+ohc* and slightly lower for *IB-2+hc*, the model with constrained backbone of transmembrane helices. **(C)** RMSD for the transmembrane helix bundle of each dodecamer subunit with respect to its initial structure (0 ns). The values for all 60 subunits are shown in light gray. Mean RMSD of the 12 helix bundles of each dodecamer model variant is shown in bold. The values were relatively constant after ~ 70 ns indicating convergence and were similar for IB-1, IB-2, IB-2+lic and IB-2+ohc and slightly lower for IB-2+hc.

Supplementary analysis of positioning of the β3β4 loop close to the pore center

Inspecting the CLDN10b and CLDN10a models, we observed variations in the conformation and the positioning of the β3β4 loop. In one variant, a residue at the loop tip (L56/T58 in CLDN10a/-10b) was sticking into the β1β2 loop of a *cis*-interacting subunit (loop-in-loop variant, *lil,* **Figure S5D left**). In the other variant, the two loops were also close but not inserted into each other (non-inserted variant, *ni,* **Figure S5D right**). The appearance of both variants in one dodecamer contributed to partial asymmetry of the models. Thus, for the CLDN10a dodecamer models, we aimed to reduce the partial asymmetry by modeling dodecamer starting structures for the simulations containing only one of the two variants. We concentrated on the *lil* variant for the following reasons:

(i) It decreased the solvent accessibilityof the hydrophobic L56, (ii) for the *lil* variant in CLDN10b, T58 was close to S33 in β1β2 loop (**Figure S5D left**, **6E (iii))**. Sequence comparison of the channel-forming CLDN2, -10a, -10b and -15 (**Figure S5C)** led to the hypothesis that the conserved polar residue corresponding to S33 in CLDN10b interacts either with the polar residue corresponding to T58 in CLDN10b (T59 in CLDN2) or if no polar residue is present at this position (L56 in CLDN10a, L57 in CLDN15) with a polar residue at the position corresponding to T41 in CLDN10a (N42 in CLDN15). Thus, the *lil* variant appeared consistent with the sequence covariations among claudins.

For most β1β2 loop – β3β4 loop interfaces of the CLDN10a dodecamer model variants, the *lil* variant was maintained during the simulations, resulting in close L56-T31 mean distances (**Figure 4C).** However, the *ni* variant was also found at individual interfaces (**Figure S5D right**, **S5E (ii)).** In sum, the data indicate that the *lil* variant can support stability and symmetry of CLDN10a oligomers.

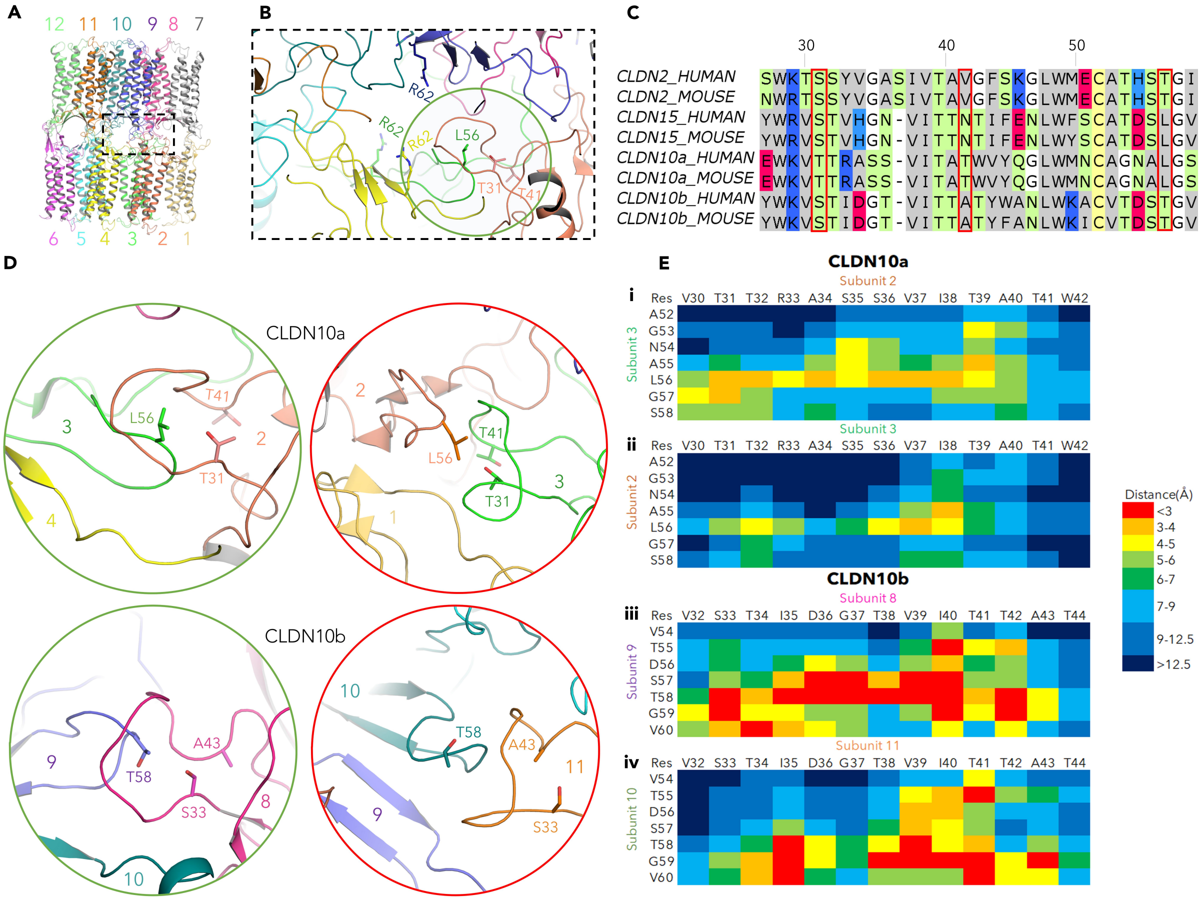

Figure S5. Positioning of β3β4 loop close to the pore center and *cis*-contact with β1β2 loop of neighboring subunit. **(A)** Overview of CLDN10a-*IB-2* dodecamer. **(B)** Close-up of dashed black box showing *cis*-contact region. Claudin subunits shown as colored cartoons with relevant residues as sticks. **(C)** Sequence alignment of region from β1-strand to beginning of β4-strand for channel-forming CLDN2, -10a, -10b and -15. Numbers corresponding to CLDN10a. Positions of polar residues getting in close proximity in CLDN10 model variants are marked with red boxes. Covariation of these residues between claudins is in line with polar interaction between two out of three residues for each claudin subtype. **(D)** Close-up of green circle in (B) with *cis*-contacts of β3β4 loop with β1β2 loop. Two different variants are shown (left, loop-in-loop (*lil*) variant; right, non-inserted (*ni)* variant) for CLDN10a (top) and CLDN10b (bottom). In the variants depicted on left, polar interactions between two of the three covarying residues are better formed (CLDN10a T31-T41, CLDN10b S33-T58). **(E)** Contact maps of β1β2 loops (30/32 - 42/44) with β3β4 loops (52/54 – 58/60) for CLDN10a and CLDN10b and two different variants. For CLDN10a, the *lil* variant 2/3 (i) shows lower distance for T31-L56 than that the *ni* variant 3/2 (ii). For CLDN10b, the *lil* variant 8/9 (iii) shows lower distance for S33-T58 than the *ni* variant 11/10 (iv).

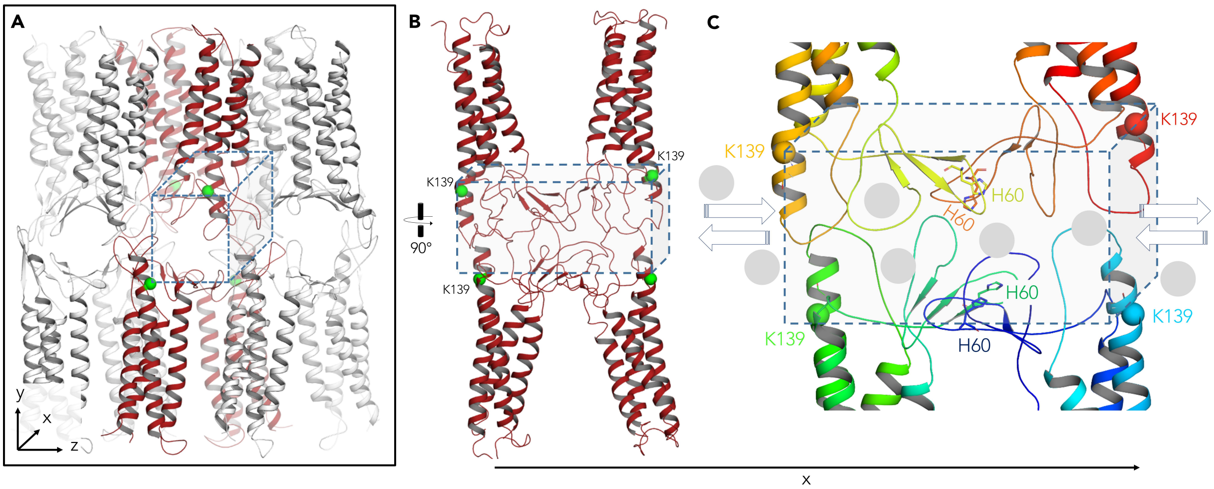

Figure S6. Representation of the imaginary cuboid used for the ion displacement calculation. The cuboid is represented with blue dotted lines. **(A)** Overview of cuboid in CLDN10a dodecamer model, with the Cα atoms of the K139 residues (K141 in CLDN10b), that serve as the boundaries of the cuboid, represented as green spheres. **(B)** Side view of the cuboid. **(C)** A zoomed-in view of the cuboid. H60 tetrad at the center of the pore (represented as sticks) serves as the bottleneck for an ion to be considered passing the pore. Gray spheres represent the ions passing through the pore.

Video S1. Video overview of Cldn10a dodecamer model (IB-2) with face-to-face- and linear-cis-interfaces as well as β1β2 loop tip clusters shown.

Video S2. A video overview of ion movement through Cldn10a channel in one of the applied electric field simulations (-0.8 V). Relevant residues in the pore are represented as sticks. Salt bridges formed by these residues with the Cl- ions are shown as dotted magenta lines. Starting at 00:23 timestamp, for a single Cl- ion, represented as a blue sphere, movement through the pore can be seen. In the 2nd part of the video the aspect ratio (width/height) is changed for technical reasons.

1. Nagarajan, S.K.; Klein, S.; Fadakar, B.S.; Piontek, J. Claudin-10b cation channels in tight junction strands: Octameric-interlocked pore barrels constitute paracellular channels with low water permeability. *Comput Struct Biotechnol J* **2023**, *21*, 1711-1727, doi:10.1016/j.csbj.2023.02.009.
